# Preparation of Activated Carbon from Popcorn by Potassium Nitrate and Its Application in Supercapacitors

**DOI:** 10.1021/acsomega.5c03461

**Published:** 2025-06-11

**Authors:** Chih-Yung Wu, Yi-Sheng Chen, Chen-Yu Lee, Chun-Fu Huang, Yu-An Tsai, Tzu-Hsien Hsieh

**Affiliations:** † Department of Aeronautics and Astronautics, National Cheng Kung University, 1 University Road, Tainan 701401, Taiwan; ‡ International Degree Program on Energy Engineering, National Cheng Kung University, 1 University Road, Tainan 701401, Taiwan; § Institute of Space Systems Engineering, National Cheng Kung University, 1 University Road, Tainan 701401, Taiwan; ∥ Green Technology Research Institute, 83504 CPC Corporation, Taiwan, Kaohsiung 811251, Taiwan

## Abstract

The efficient and environmentally friendly preparation of activated carbon is an important issue within the field. In the present study, fluffy biomass derived from popcorn was treated as the raw material and subsequently carbonized with the addition of potassium nitrate in ambient air. It is noteworthy that this preparation method does not employ strong bases or other environmentally harmful chemicals, such as potassium hydroxide or anhydride chloride. The prepared activated carbon was subjected to a comprehensive analysis utilizing scanning electron microscopy, transmission electron microscopy, X-ray diffraction, elemental analysis, nitrogen adsorption–desorption isotherms, Raman spectroscopy, and X-ray photoelectron spectroscopy to investigate the influence of preparation parameters and environmental conditions on the physicochemical properties. The postprocessed activated carbon was deemed suitable for the fabrication of supercapacitors. The resulting supercapacitors were evaluated through cyclic voltammetry (CV), electrochemical impedance spectroscopy (EIS), and galvanostatic charge–discharge (GCD) tests to assess their electrochemical properties. The results indicated that activated carbon treated in ambient air exhibited superior surface characteristics, and the supercapacitors fabricated with this activated carbon demonstrated enhanced performance. Notably, the supercapacitor constructed with the best-activated carbon achieved the highest specific capacitance of 93.20 F/g and an energy density of 23.59 W h/kg.

## Graphical abstract

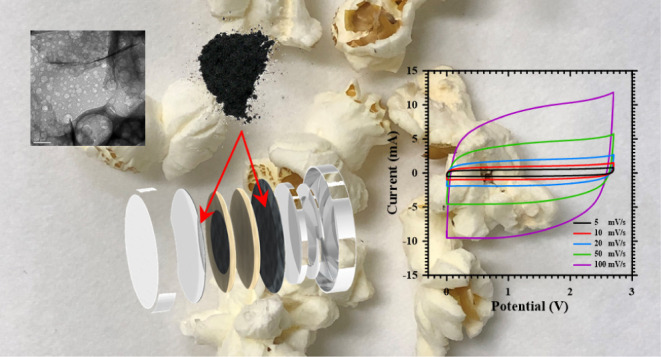

## 1 Introduction

Activated carbon is recognized as a porous carbon material characterized by low cost, stable physical and chemical properties, an adjustable pore structure, and a high surface area.^1^ It has been widely applied in the removal of heavy metals,^2,3^ dye adsorption,^4,5^ gas storage,^6,7^ material enhancement,^8,9^ and as an electrode material for supercapacitors.^10,11^ Its application as an electrode material is attributed to the presence of a rich pore structure, which facilitates charge transport and storage.^12^ The performance of supercapacitors is directly influenced by the number and size of these pores. It is well-known that supercapacitors can be a feasible energy storage method.^13^ Although the method of preparation for activated carbon is quite mature, it is still warranted for research on the process of environmental friendliness, sustainability, and performance improvement.^14^

Activated carbon features an amorphous internal structure made up of sp^2^-hybridized graphene and sp^3^-hybridized carbon components.^15,16^ Its surface includes a complex pore network, with a specific surface area typically ranging between 500 and 2000 m^2^/g. Based on the standards of the International Union of Pure and Applied Chemistry (IUPAC), the porous structure of activated carbon is classified based on pore size into three types: (I) macropores, which have diameters larger than 50 nm; (II) mesopores, with diameters between 2 and 50 nm; and (III) micropores, with diameters less than 2 nm.^17^ Significantly, mesopores facilitate electron transitions, while micropores contribute significantly to electricity storage. Structures with a higher proportion of mesopores can enhance the operational current density.^18,19^

The use of fossil fuels as precursors in the production of high-quality activated carbon is associated with environmental concerns. Consequently, the production of biochar to yield activated carbon from biomass has gained increased attention.^20^ Biomass is primarily sourced from agricultural waste and food waste.^21,22^ These precursors, which are characterized by lower carbon content, result in biochar produced through direct carbonization that also exhibits limited porosity, leading to a reduced adsorption capacity. To enhance the adsorption capacity of the products, activation reactions are typically employed to increase the material’s porosity. Activation methods can be categorized into physical activation and chemical activation, where oxidizing gases and chemical agents were utilized to interact with biochar, respectively. Various characteristics of activated carbon can be achieved by carefully adjusting process parameters, including activation agents,^23^ process temperature,^24^ process duration,^25^ and activation agents ratio.^12^ Phosphoric acid,^26^ zinc chloride,^27^ sodium hydroxide,^28^ and potassium hydroxide^29^ are the most commonly used activators in contemporary applications. These activators exhibit high corrosivity at elevated temperatures and may potentially cause environmental damage. In addition, it has been reported in the literature that carbon activation processes conducted in airflow lead to an increased specific surface area of the resultant product.^30,31^ It is noted that the traditional carbonization processes are usually performed in the inert gas environment to avoid carbon oxidation and to increase the yielding ratio.

The formation of popcorn occurs due to the vaporization of moisture within corn kernels at elevated temperatures, resulting in significant pressure that ultimately breaches the hard shell. This process causes the starch contained within to expand rapidly. The resulting expanded starch manifests as sponge-like flakes of various shapes,^32^ characterized by micron and nanometer scales. The honeycomb structure formed is particularly advantageous for creating a rich porous framework and a high specific surface area in biochar.^33^ Consequently, porous carbon derived from popcorn has been utilized in applications such as CO_2_ adsorption,^6^ dye adsorption,^33^ water purification,^34^ electrocatalysis,^35^ and electrode materials.^36,37^ Furthermore, the abundant production and low cost of corn underscore the practicality of popcorn production, which is characterized by its simplicity and speed, requiring no chemical additives and enabling large-scale output. Thus, popcorn can be recognized as an environmentally friendly and exceptional precursor for biocarbon. However, past studies usually use chemical agents, which were mentioned in the previous paragraph, to activate the popcorn-yield-activated carbon.

Potassium nitrate, recognized as a widely utilized oxidizer in the production of propellants,^38^ fireworks, smoke bombs, flares, and other products, has been identified as an effective agent for the preparation of activated carbon in a single step. During the pyrolysis process of potassium nitrate, a sequential transformation occurs, progressing from KNO_3_ to KNO_2_, then to KNO, and ultimately to K_2_O. In the context of carbon torrefaction, the interaction between steam and volatile elements is noted to significantly contribute to the formation of micropores, with potassium nitrate supplying both gaseous byproducts and potassium atoms. The role of potassium atoms in the activation process of carbon has been duly acknowledged in prior research.^39,40^ In earlier studies, it was demonstrated that the preparation of activated carbon could be achieved through the mixing of potassium nitrate and liquefied glucose. The resulting activated carbon has been employed in the fabrication of supercapacitors.^41^ In summary, a process has been developed that employs the mixing of potassium nitrate with raw biomass material for the purpose of precarbonization, followed by the treatment of the resultant products in a muffle furnace for calcination and activation. This method is characterized as a single-step procedure for the preparation of activated carbon, distinguishing it from conventional potassium hydroxide (KOH) activation procedures, which are recognized as a two-step method. The traditional process typically involves torrefaction, subsequent mixing with KOH, and high-temperature calcination for activation. In comparison to potassium hydroxide, potassium nitrate is regarded as a safer alternative. It has been established that the initial stage of torrefaction, followed by the cooling of the mixed potassium hydroxide, can be omitted in the preparation process. However, concerns have been raised regarding the efficacy of potassium nitrate in a single-step activation process when the mixing is performed at the powder level, as opposed to when liquefied glucose is utilized. In the present study, popcorn was selected as the biomass precursor. The precursor was subjected to pulverization and subsequently mixed with potassium nitrate powder to undergo torrefaction and activation. The resultant products, varying under different parameters, were utilized in the fabrication of supercapacitors, and their performance was rigorously evaluated.

## 2 Methodologies and Apparatus

### 2.1 Preparation of the Activated Carbon

The preparation procedure of the activated carbon is depicted in Figure 1, with the corresponding experimental details in Table 1. The presence of additives such as fat, sugar, and salt in commercial popcorn renders it unsuitable as a raw material for the current study. Consequently, it is necessary for popcorn to be prepared in a laboratory setting. The dried maize was evenly sprinkled on the stainless tray and subsequently placed in a preheated oven at a temperature of 200 °C. During production, the oven temperature must be maintained between 190 and 210 °C to ensure that most corn kernels achieve complete bursting.^32^ The popcorn was to remain in the oven for 5–8 min before being removed. The prepared popcorn was to be transferred into a grinder, where the crushing process was initiated, and the process ran for 5 min to ensure that all popcorn was converted into powder. Upon completing the crushing, the popcorn powder was stored in a sealable container. Additionally, the popcorn powder and potassium nitrate must be dried at a temperature of 110 °C overnight before the next step.

**Figure 1:**
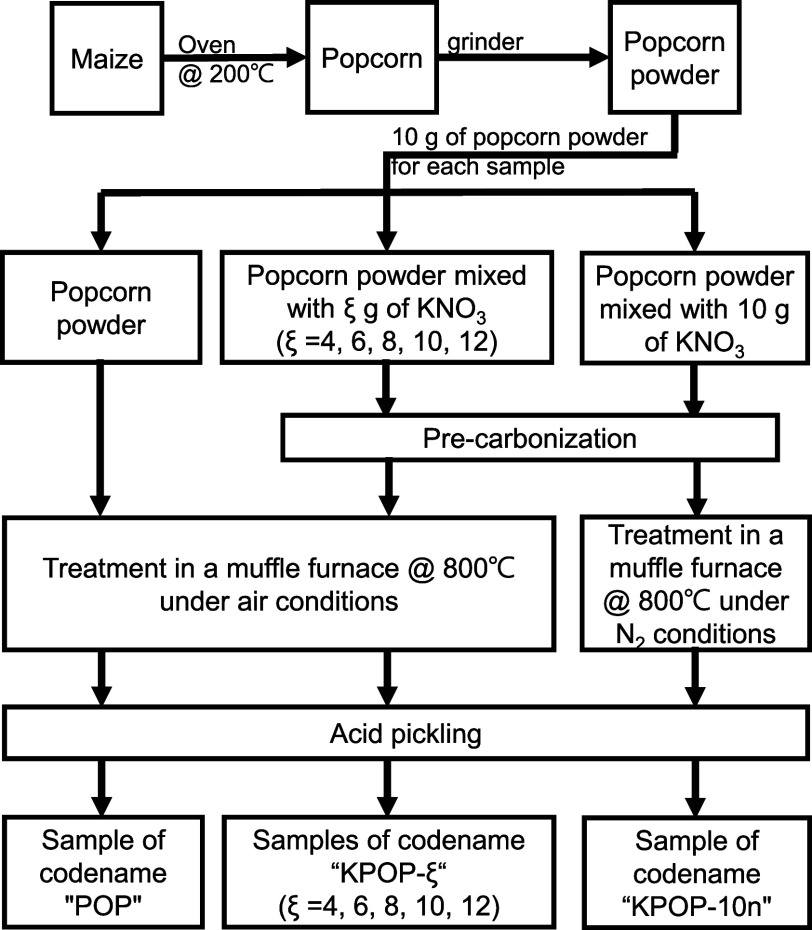
Procedures for the preparation of activated carbon samples.

**Table 1:** Experimental Cases

Case no.	Compositions (% wt) Popcorn/KNO_3_	pretreatment	Calcination (gas stream)	Post-treatment
POP	100/0	Hull-rupturing/Grinding/mixing	800 °C (air)	Pickling
KPOP-4	10/4	Hull-rupturing/Grinding/mixing	800 °C (air)	Pickling
KPOP-6	10/6	Hull-rupturing/Grinding/mixing	800 °C (air)	Pickling
KPOP-8	10/8	Hull-rupturing/Grinding/mixing	800 °C (air)	Pickling
KPOP-10	10/10	Hull-rupturing/Grinding/mixing	800 °C (air)	Pickling
KPOP-10*n*	10/10	Hull-rupturing/Grinding/mixing	800 °C (N_2_)	Pickling
KPOP-12	10/12	Hull-rupturing/Grinding/mixing	800 °C (air)	Pickling

For each sample, 10 g of popcorn powder was measured and mixed with potassium nitrate powder in varying weights (4, 6, 8, 10, and 12 g). The mixtures were placed into a ball mill and subjected to mixing at a constant speed of 230 rpm for 2 h. Upon completion of the milling and mixing process, 1 g of the resultant mixture was extracted and placed into an alumina crucible. The powder was then ignited using a gas torch to induce an oxidation–reduction reaction between the popcorn powder and potassium nitrate to achieve the purpose of precarbonization. The molecular formula of the feedstock utilized in the study, accompanied by the respective suppliers, is provided in Table 2.

**Table 2:** Chemical Reagents

Chemical reagents	Formula	Supplier
Activated carbon		
Dried maize	-	PopSmile Co. Ltd.
Potassium nitrate	KNO_3_	Scharlab
Hydrogen chloride acid	HCl	Acros
Nitrogen	N_2_	YunHai Gas Co. Ltd.
Supercapacitor		
Methyl methacrylate (MMA)	C_5_H_8_O_2_	TCI
Ethyl acetate	C_4_H_8_O_2_	Fisher
Carbon black, acetylene	C	Alfa
TEABF_4_/PC (1M)	C_8_H_20_BF_4_N	ACROS/ORGANI
Argon	Ar	YunHai Gas Co. Ltd.

The samples were treated in a muffle furnace under a stable air flow (7.5 L/min) for the calcination. The samples were heated from room temperature to 800 °C at a heating rate of 10 °C/min and maintained at this temperature for 2 h to facilitate the removal of residual volatile matter. Then, the samples were slowly cooled down to ambient temperature. The samples were subjected to washing with 35% hydrochloric acid for 30 min to eliminate impurities and unreacted potassium nitrate. The pickled sample was thoroughly rinsed with ample deionized water to ensure the neutrality of the wastewater. The washed sample was then dried for 12 h at 110 °C until completely dry. The products were marked as KPOP-ξ, where ξ indicates the weight of potassium nitrate added per 10 g of popcorn powder. It is noted that a sample denoted as KPOP-10n was calcined in the muffle furnace under a stable N_2_ flow. The preparation procedures are the same as those under a stable airflow. Moreover, a control sample denoted as POP was calcinated without mixing with potassium nitrate for precarbonization.

### 2.2 Fabrication of the Supercapacitor

Similar to the process proposed in the previous study.^41^ The fabrication of the supercapacitor was conducted using the 2032 kit, a button battery specification characterized by a diameter of 20 mm and a height of 3.2 mm, as illustrated in Figure 2. Notably, the kit was comprised of multiple components, including an upper case, a spring, a spacer, a positive electrode, cellulose membranes with attached activated carbon, and a lower case. The electrodes, both positive and negative, were fabricated from conductive carbon paper. It is worth mentioning that activated carbon was prepared through various processes, specifically KPOP-8, KPOP-10, KPOP-10n, and KPOP-12, for the construction of the supercapacitors.

**Figure 2:**
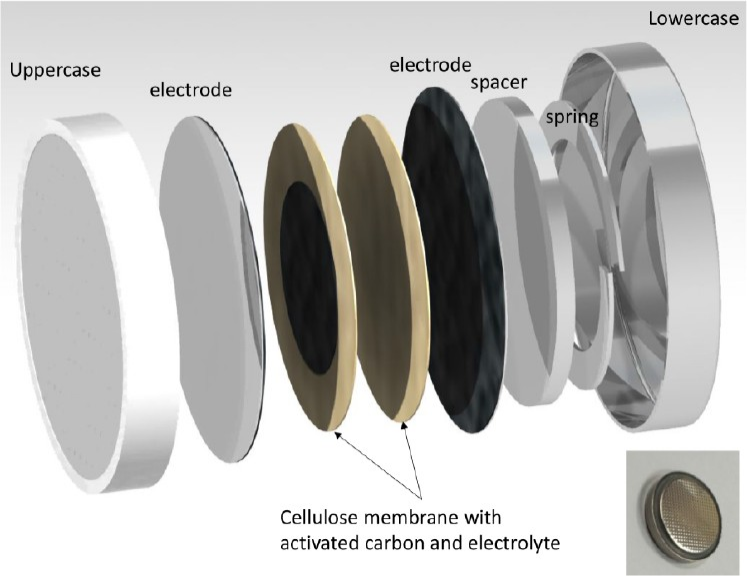
Components of the 2032 button battery kit.

Prior to utilization, the activated carbon was subjected to drying at 110 °C for a duration of 24 h to ensure the complete evaporation of any residual moisture. A mixture consisting of activated carbon, carbon black, and MMA in a ratio of 1:0.2:0.1 was combined with an equal weight of ethyl acetate in test tubes. Additionally, acetylene black, recognized for its distinguished physical properties, was incorporated into the mixture to enhance conductivity. The resultant blend was subjected to uniform mixing in an ultrasonic shaker for 15 min, followed by oscillation in an ultrasonic oscillator for an additional 30 min. Subsequently, the mixed solution was deposited onto a cellulose membrane, which was positioned atop filter paper in a suction filtration device. This process facilitated the even distribution of activated carbon on the membrane, which was subsequently dried at 110 °C for 24 h.

It is noteworthy that the assembly of cellulose membranes was conducted within a glovebox that was purged of moisture and filled with argon to create an inert atmosphere. During the assembly process, 1 M TEABF4/PC electrolyte was applied to the activated carbon-coated cellulose isolation film to ensure uniform moistening. The final assembly, illustrated in Figure 2, involved sequential stacking of various components of the 2032 kit, including a lowercase, a spring spacer, two 1 μm-thick electrodes, two cellulose membranes, and the uppercase. It was sealed utilizing a sealing machine. Furthermore, the chemical reagents and consumable materials essential for the assembly of supercapacitors were also itemized in Table 2.

### 2.3 Apparatus

The collected activated carbon underwent a series of detailed analytical procedures to evaluate its purity and properties. The surface morphology was examined with precision using a JEOL JSM-7000 model field-emission scanning electron microscope (SEM). An ultrahigh-resolution transmission electron microscope, specifically the JEOL JEM-2100F CS STEM model, was employed to investigate the nanometer-scale structure of the samples. X-ray diffraction (XRD) analysis was conducted using a D8 Advance X-ray diffractometer from Bruker to characterize the crystalline structure of the samples. The BET method was applied using an ASAP 2020 PLUS apparatus to analyze the surface area properties of the activated carbon. Elemental and proximate analyses were also performed to assess the purity of the prepared activated carbon. An elemental analyzer (VARIO EL CUBE) was utilized for the elemental analysis, which provided insights into the composition of the samples, including carbon, hydrogen, oxygen, nitrogen, and sulfur. The findings from the elemental analyses contributed significantly to evaluating the sample’s purity. Based on the Raman effect, Raman spectrometry was employed to assess the surface chemical properties, structure, and degree of order of the carbon materials. For this purpose, the Renishaw Ramascope 2000 was utilized. X-ray Photoelectron Spectroscopy (XPS) was employed to examine the functional groups on the surface of the activated carbon using the ULVAC-PHI VersaProbe 4 instrument.

The fabricated supercapacitors were subjected to a comprehensive series of analyses, including cyclic voltammetry (CV), electrochemical impedance spectroscopy (EIS), and galvanostatic charge/discharge testing. Cyclic voltammetry, an essential technique in electrochemical research, was performed using the Maccor Model 4200M. Scan rates of 5, 10, 20, 50, and 100 mV/s were employed, and the voltage span ranged from 0 to 2.7 V. The CV measurement involved a rapid voltage scan that reversed direction, resulting in a CV curve that elucidated the charging and storage mechanisms as well as the performance of the supercapacitors. An ideal CV curve for a supercapacitor is characterized by a nearly rectangular shape.^43,44^

Electrochemical impedance measurements were conducted using an electrochemical workstation (VersaSTAT3), with frequencies varying from 0.1 MHz to 0.1 Hz, accompanied by a small fixed sine wave voltage of 10 mV. The resulting Nyquist plot offered insights into the internal resistance and the resistance associated with the diffusion layer.^45^ The charge and discharge capacitance, along with the cycle life, were evaluated through galvanostatic charge/discharge techniques. The complete charge was measured across various current densities, ranging from 0.5 A/g to 5 A/g, over the voltage range of 0 to 2.7 V, while the discharge process was conducted from 2.7 to 0 V. The analysis relied on plots of voltage versus time. Typically, a supercapacitor displays a symmetrical linear triangular profile in its capacitance characteristics.^46^ It was observed that the specific capacitance value diminishes with increasing current density, attributed to the diffusion limitations of electrolyte ions entering the minuscule pores under elevated current densities.

## 3 Results and Discussions

### 3.1 Activated Carbon Yield Rate

As shown in Figure 3, the analysis of ground popcorn powder, the mass fraction of carbon, oxygen, nitrogen, and hydrogen were determined to be 42.42%, 49.34%, 1.93%, and 6.313%, respectively. Following the carbonization process, where potassium nitrate was not added, the proportions of these elements in the resulting popcorn samples were found to change significantly, with carbon content increasing to 78.76%, while oxygen, nitrogen, and hydrogen decreased to 16.50%, 3.16%, and 1.578%, respectively. The samples carbonized with the addition of potassium nitrate exhibited a carbon content ranging from 65% to 74%, with an observed increase correlating with the quantity of potassium nitrate incorporated into the raw material. It should be noted that adding more than 12% potassium nitrate will result in extremely low yields. Therefore, activated carbon cannot be prepared by adding excess potassium nitrate. A comparative analysis of KPOP-10 and KPOP-10*n* revealed that KPOP-10*n* possessed a higher carbon content than KPOP-10, a difference attributed to the protective effects of nitrogen during the process. Furthermore, a comparison between KPOP-10*n* and the original POP sample indicated that, after activation, the increase in oxygen content was only 2%, suggesting that the majority of this increase during the activation process resulted from exposure to air.

**Figure 3:**
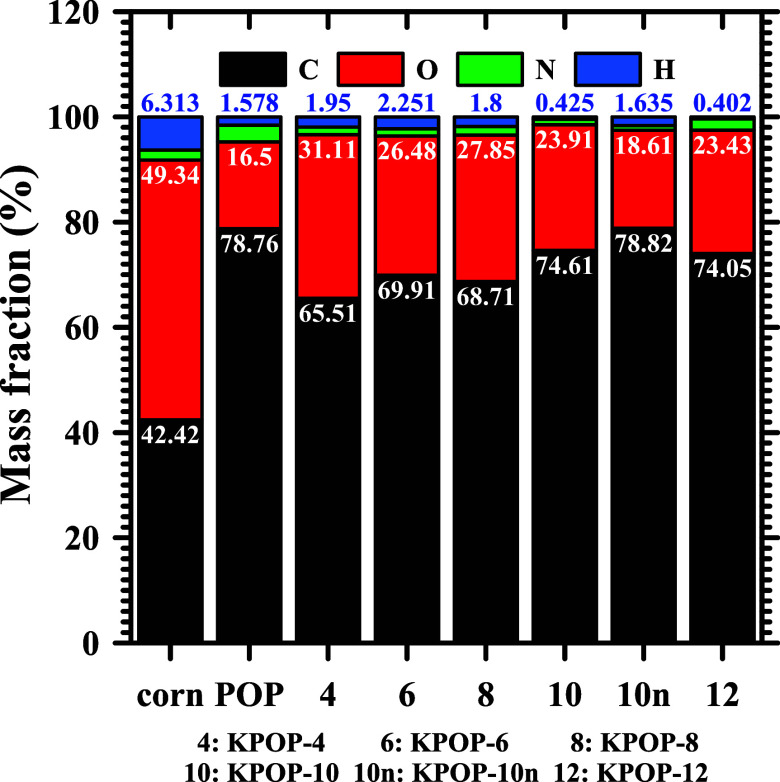
Ultimate analysis of products.

The yield is recognized as a crucial factor for evaluating the feasibility of activated carbon production processes. It is determined by utilizing eq 1,^47^ where *m*_ac_ and *m*_b_ are mass of the activated carbon product, and the initial mass of popcorn powder, respectively. Additionally, the carbon conversion rate, calculated through eq 2, where *m*_ac_, *m*_b_, *Y*_c*/*ac_, and *Y*_c*/*b_ are the mass of the activated carbon product, the initial mass of popcorn powder, the mass fraction of carbon atom in the activated carbon product, and the mass fraction of carbon atom in the popcorn powder, respectively, serves as an important analytical parameter. In the present study, the denominator for the carbon conversion rate is established based on the carbon content in popcorn powder. The carbon content of popcorn powder was measured at 42.42%, as shown in Figure 3. It is imperative to ascertain the proportion of carbon that may be transformed into CO or CO_2_ gas and subsequently lost during the course of the process,^48^ or that may be converted into volatile compounds such as methane during later stages of high-temperature calcination (600–800 °C).^49^

1 $$\chi_{y}=\frac{m_{ac}}{m_{b}}\times 100\%$$

2 $$\chi_{cc}=\frac{m_{ac}\times Y_{c/ac}}{m_{b}\times Y_{c/b}}\times 100\%$$

Figure 4 illustrates a bar chart that represents the yield and carbon conversion rate under various experimental conditions labeled as POP, KPOP-4, KPOP-6, KPOP-8, KPOP-10, KPOP-10*n*, and KPOP-12. It was observed that the preparation process executed without the addition of potassium nitrate resulted in the highest values for both the yield and the carbon conversion ratio. A decreasing trend was identified among KPOP-4, KPOP-6, KPOP-8, KPOP-10, and KPOP-12, with the carbon conversion rate consistently exceeding the yield. KPOP-10*n* was noted to exhibit a slight increase in comparison to KPOP-10, yet it remained lower than KPOP-8. Additionally, it was emphasized that during the activation process of KPOP-10, protection by nitrogen gas was employed to prevent the involvement of oxygen in the reaction, which significantly enhanced both the yield and carbon conversion rate when compared to KPOP-10.

**Figure 4:**
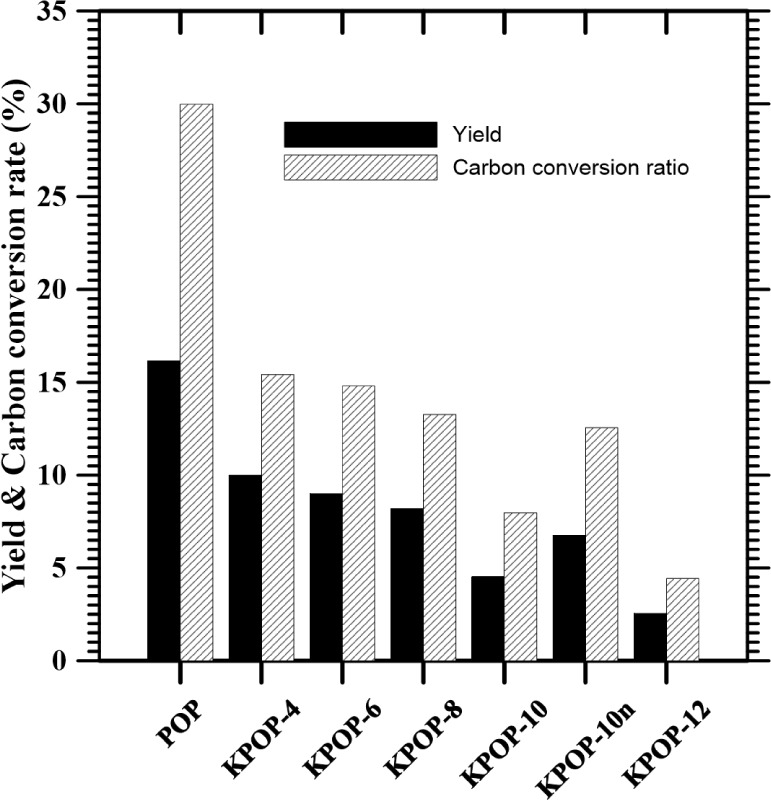
Yield and carbon conversion rate.

### 3.2 Microstructure of the Activated Carbon

As illustrated in Figure 5, the scanning electron microscope (SEM) image of a sample magnified 5000 times reveals a smooth surface devoid of visible pores for the case of POP. In comparison, samples subjected to activation exhibit a more fragmented structure characterized by a uniform and dense distribution of pores across the surface. It was observed that with an increasing quantity of potassium nitrate, both the density and size of the pores also increase. The SEM analysis confirmed that the presence of potassium nitrate facilitates the formation of pores within the sample during the activation reaction, and it was noted that the structural changes correlate with the concentration of the potassium nitrate. As shown in Figure 6, comparing the SEM picture of activated carbon prepared in the ambient air with that of activated carbon in nitrogen gas, it is difficult to distinguish their differences, and further examination was required.

**Figure 5:**
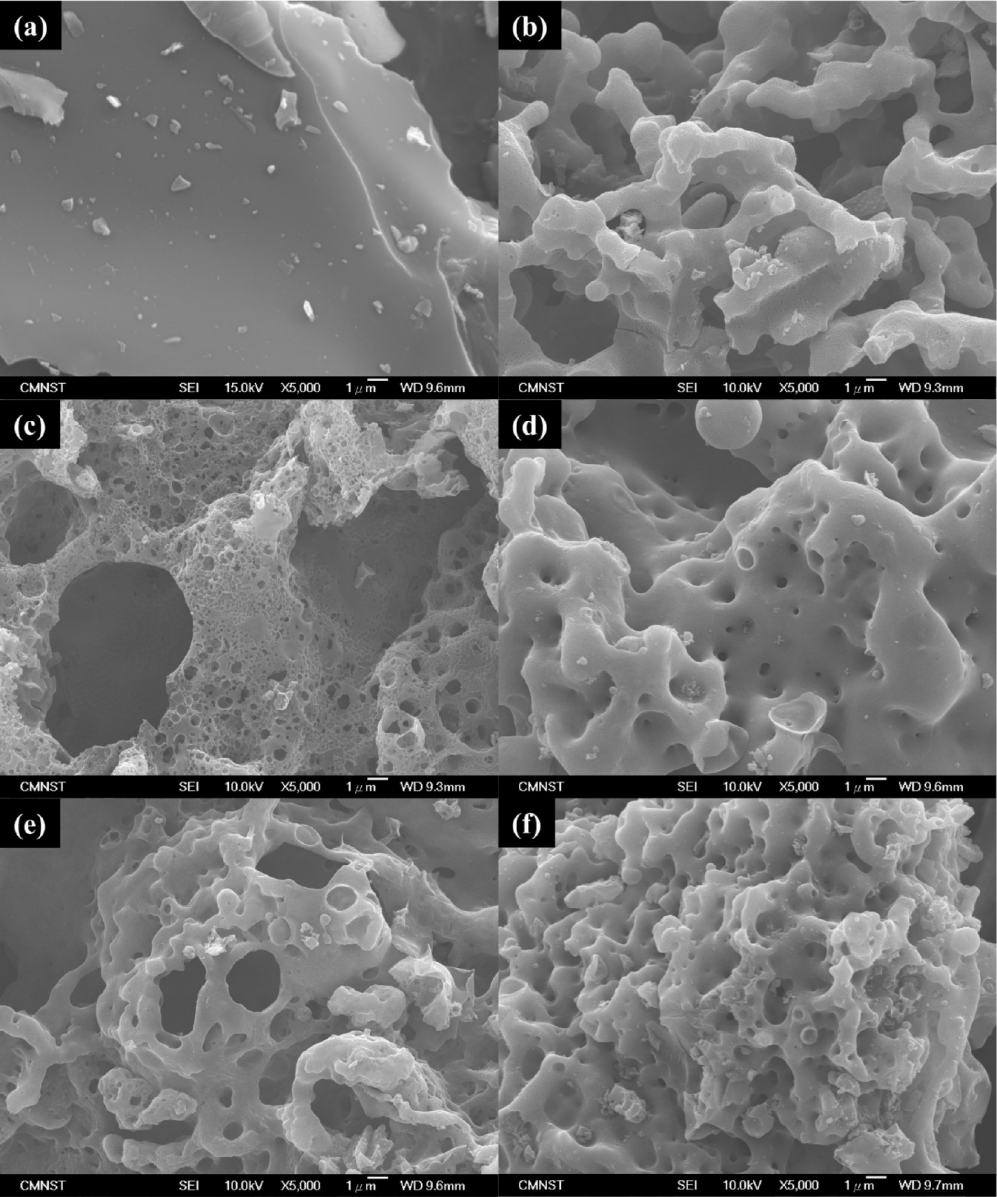
SEM images of case: (a) POP, (b) KPOP-4, (c) KPOP-6, (d) KPOP-8, (e) KPOP-10, and (f) KPOP-12.

**Figure 6:**
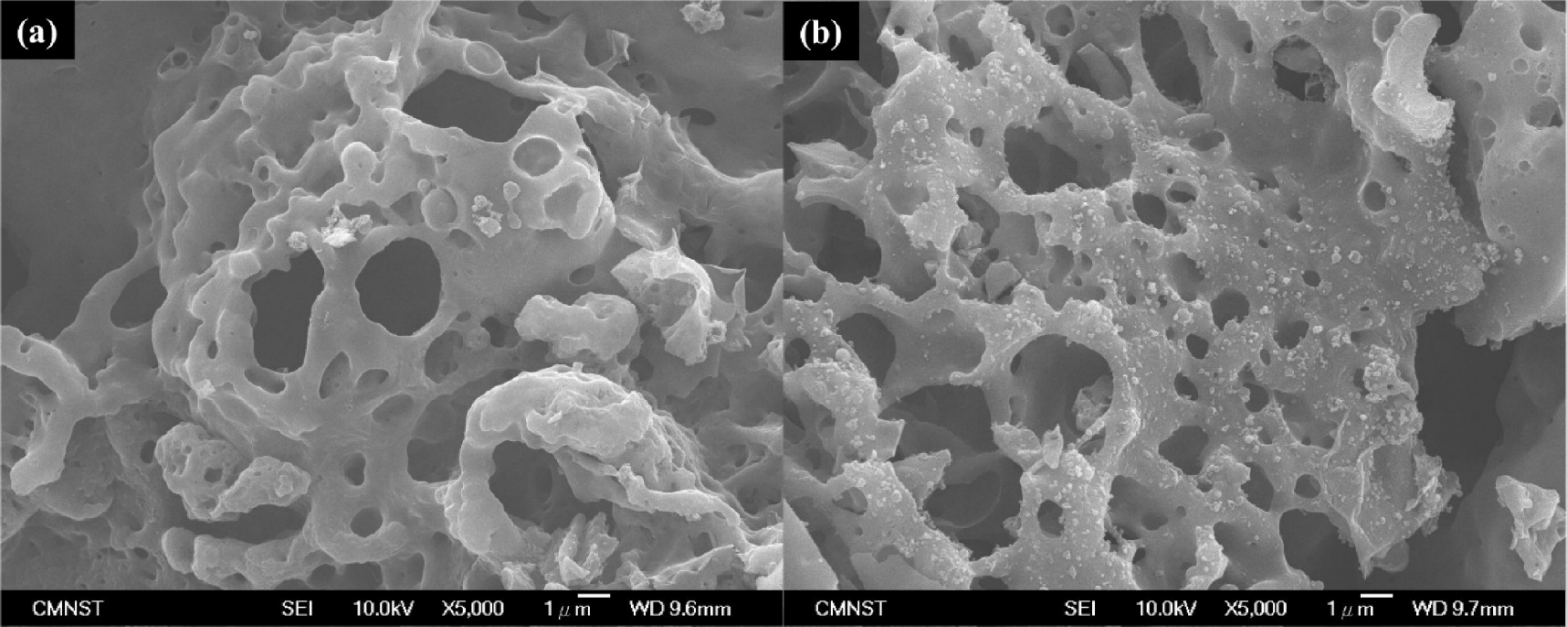
SEM images of case: (a) KPOP-10 and (b) KPOP-10*n*.

The high-resolution TEM images of the activated carbon samples, specifically POP, KPOP-4, KPOP-6, KPOP-8, KPOP-10, and KPOP-12, are presented in Figure 7. It is observed that all activated carbon samples exhibit a flake-like structure characterized by a notable distribution of pores. The majority of the samples produced in this study are found to display an irregular flake structure, in contrast to the predominantly spherical morphology of acetylene combustion soot.^42^ It is hypothesized that the carbon flakes may originate from the original spongy flakes of the popcorn, as well as from the vigorous reaction between potassium nitrate and biomass. The incorporation of potassium nitrate results in an even distribution of holes across the surface of the carbon sheets. Furthermore, it is noted that the density of pores on the carbon sheets increases with the proportion of potassium nitrate. The pore diameters of all activated samples are largely found to fall within the range of 10–50 nm, categorizing them as mesopores. This structure can facilitate the rapid diffusion of electrolyte ions during rapid charge and discharge processes.^50^

**Figure 7:**
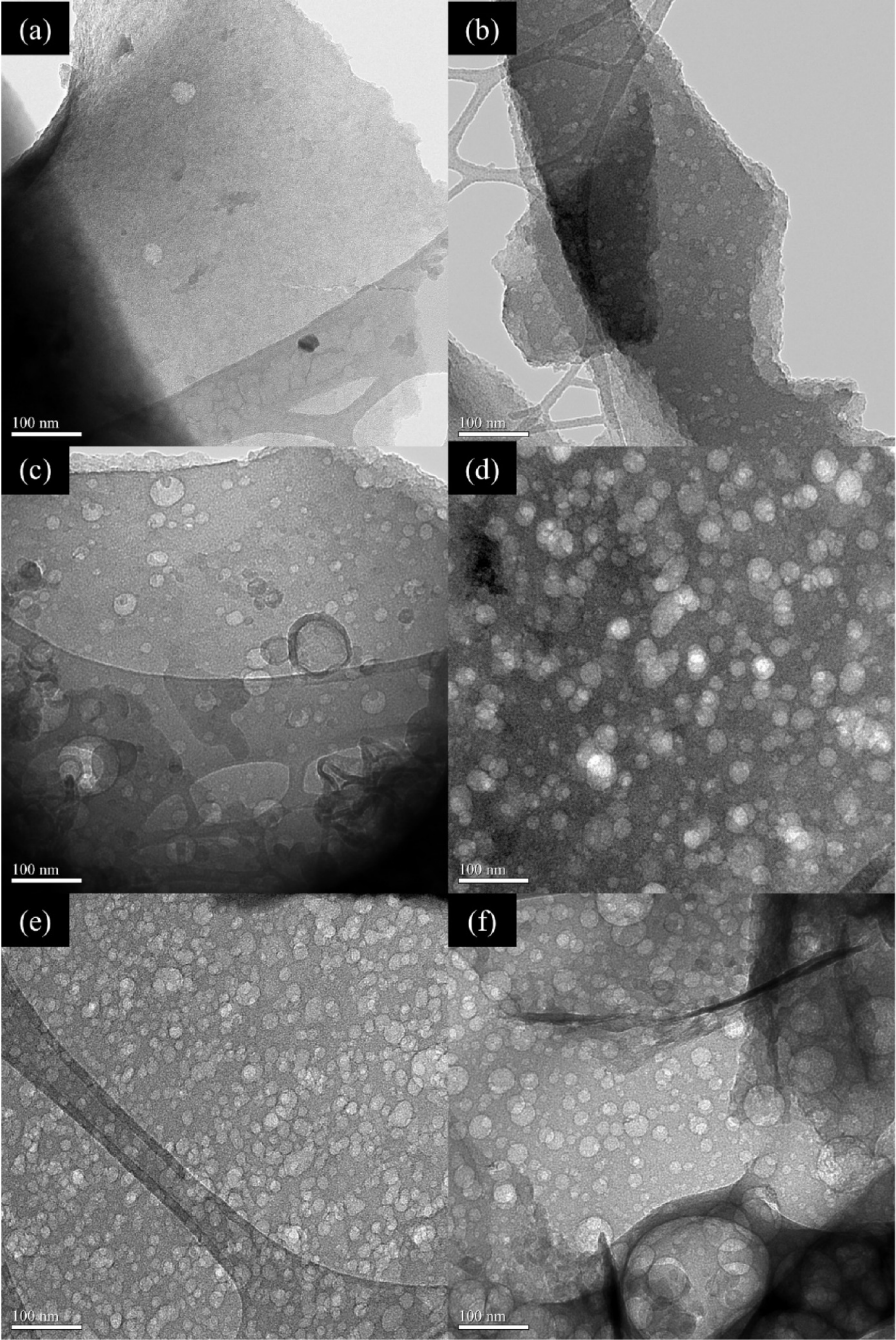
TEM images of case: (a) POP, (b) KPOP-4, (c) KPOP-6, (d) KPOP-8, (e) KPOP-10, and (f) KPOP-12.

As shown in Figure 8, similar to comparing SEM results, it is difficult to distinguish the difference between activated carbon prepared in environmental air and that prepared in nitrogen gas by comparing their TEM images, as both possess high density and uniform mesopore distribution.

**Figure 8:**
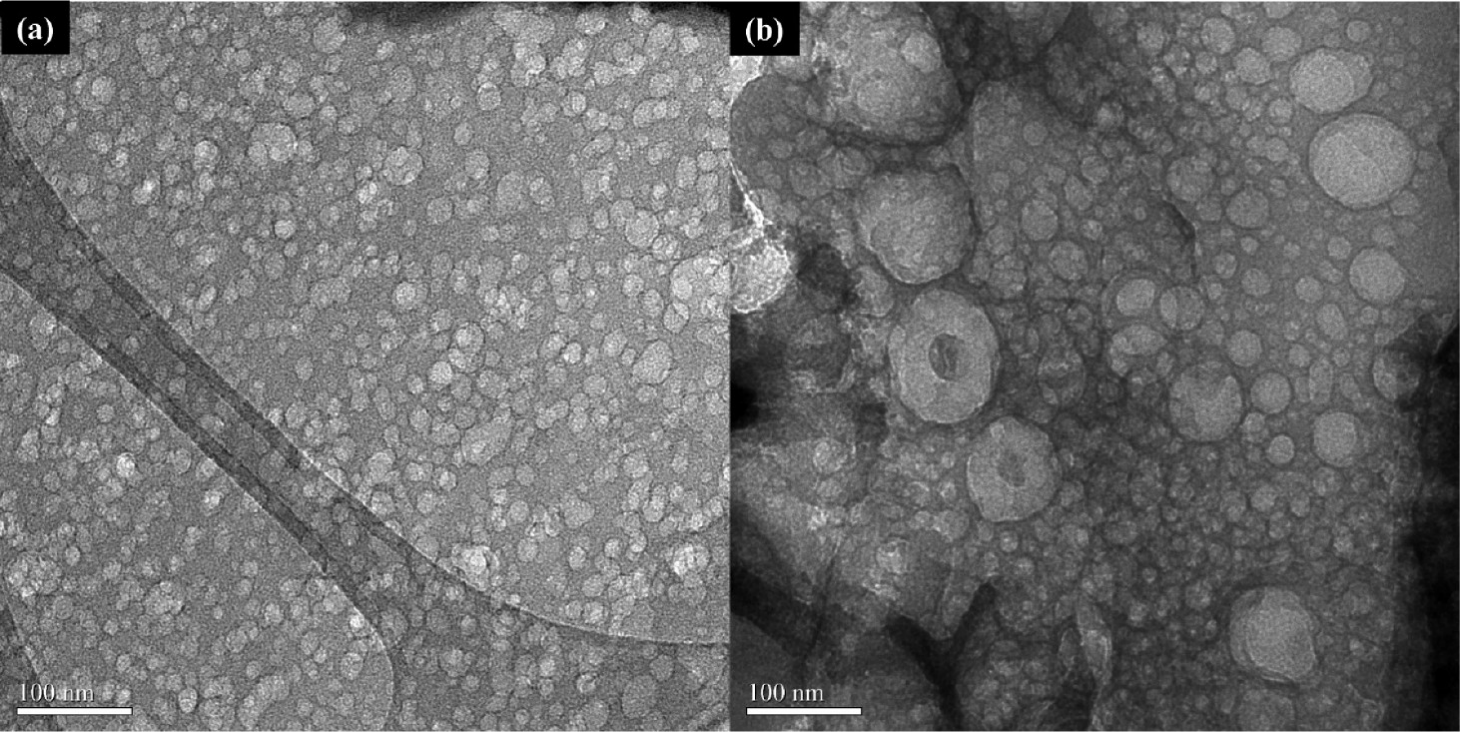
TEM images of case: (a) KPOP-10 and (b) KPOP-10n.

### 3.3 Surface Area and Pore Characteristics of Activated Carbon

The adsorption–desorption curve plots of BET measurement for each product are presented in Figure 9a, where it is demonstrated that the gas adsorption amount of the samples increases with the increasing ratio of potassium nitrate. Figure 9b illustrates the results obtained by incorporating the same proportion of potassium nitrate under varying atmospheric conditions. It is observed that further reaction of activated carbon with air is limited in a nitrogen environment, resulting in a lower generation of pores compared to those in air-activated samples. All products are characterized by a Type “I” adsorption curve, and a tendency is noted for the inflection point to shift toward a higher relative pressure region as the potassium nitrate ratio increases. This observation indicates that a greater amount of potassium nitrate contributes to the expansion of the pore diameter of the activated carbon. Additionally, the presence of an “H4” type hysteresis loop further substantiates the notion that the product exhibits a broader distribution of pore size.^51^

**Figure 9:**
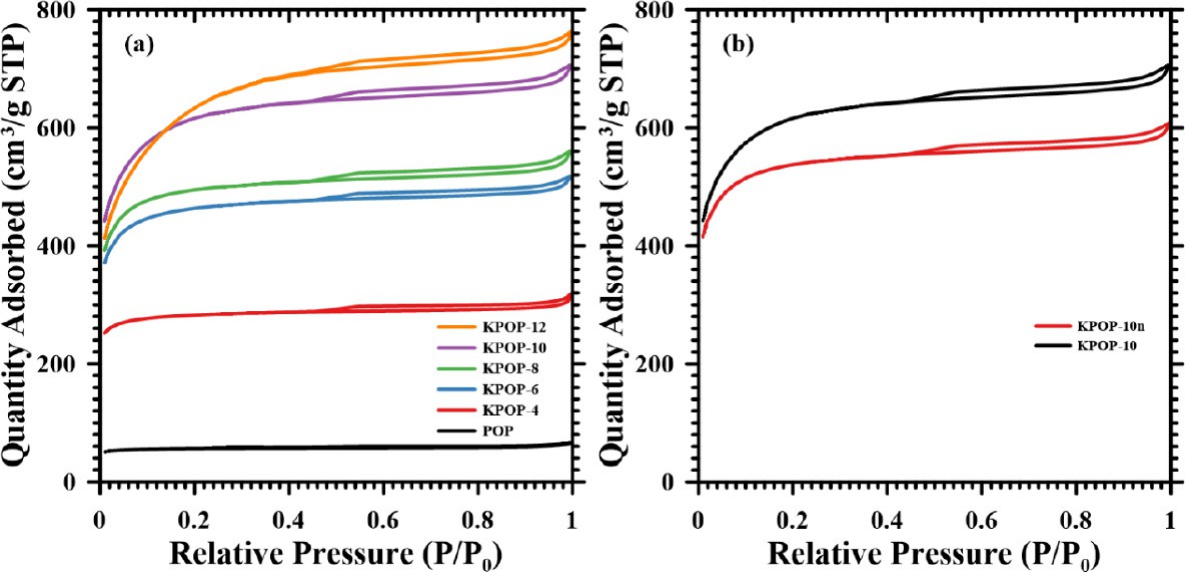
Adsorption–desorption curve plots of BET measurement: (a) POP, KPOP-4, -6, -8, -10 and -12; (b) KPOP-10*n* and -10.

The results presented in Table 3 illustrate the textural properties of various samples, including BET surface area, total pore volume, average pore width, and mesopore volume fraction in the second to fifth columns. A comparative analysis of the data for all samples treated in an air ambient reveals that the sample designated as POP exhibits the lowest BET surface area (196 m^2^/g) and total pore volume (0.149 cm^3^/g). In contrast, the incorporation of potassium nitrate is shown to enhance these properties during the preparation process significantly. It is observed that both surface area and pore volume increase progressively with the addition of potassium nitrate, reaching a maximum at KPOP-12, which records values of 2253 m^2^/g and 1.574 cm^3^/g, respectively. The average pore width remains relatively stable, oscillating between 1.961 and 2.097 nm. Notably, the mesopore volume fraction does not adhere to a strictly increasing trend; KPOP-10 (11.70%) and KPOP-12 (27.93%) are found to exhibit substantially higher values than the other samples. This observation suggests that although surface area and total pore volume generally increase with the amount of potassium nitrate utilized, mesopore volume fraction appears to be more sensitive to processing conditions, particularly at elevated concentrations of potassium nitrate. Furthermore, in the cases of KPOP-10 and KPOP-10n, it can be seen that the presence of oxygen in the air exacerbates pore size expansion, resulting in the average pore width of KPOP-10 being greater than that of KPOP-10n, and the mesopore volume fraction being elevated.

**Table 3:** Surface Area, Total Pore Volume, Averaged Pore Width, Mesopore Volume Fraction, Micropore Surface Area, Micropore to BET Surface Area Ratio, Micropore Volume, and the Micropore Volume Fraction

Cases no.	BET surface area (m^2^/g)	Total pore volume (cm^3^/g)	Average pore width (nm)	Mesopore volume fraction (%)	Micropore surface area (m^2^/g)	Micropore to BET surface area ratio	Micropore volume (cm^3^/g)	Micropore volume fraction (%)
POP	196	0.149	2.055	10.01	189	0.96	0.083	56
KPOP-4	992	0.756	1.993	3.91	943	0.95	0.416	55
KPOP-6	1632	1.227	1.961	5.67	1505	0.92	0.662	54
KPOP-8	1743	1.299	1.989	5.09	1505	0.86	0.671	52
KPOP-10	2176	1.528	2.007	11.70	2030	0.93	0.855	56
KPOP-10*n*	1894	1.381	1.984	5.71	1746	0.92	0.768	56
KPOP-12	2253	1.574	2.097	27.93	1749	0.77	0.773	49

The detailed information regarding micropore properties is subject to further analysis and estimation through the utilization of T-plots.^52^ The statistical thickness is calculated using eq 3, with the relative pressure (*p*/*p*_0_) being confined to the range of 0.2 to 0.5, which is situated above the micropore filling region and below the capillary condensation section. It is noted that *p* and *p*_o_ are the equilibrium pressure and saturation vapor pressure of the adsorbate gas. The relationship between the quantity of adsorption and statistical thickness for POP, KPOP-4, -6, -8, -10, and -12 is presented in Figure 10, while the comparison between KPOP-10 and KPOP-10*n* is depicted in Figure 11. It is noted that the data points within the specified range of 0.2 to 0.5 are marked with blue symbols and are employed for linear fitting. The *y*-intercept obtained represents the micropore volume, while the slope of the fitted line is indicative of the external surface area after being multiplied by 15.47 (nitrogen @ 77 K).

3 $$\xi \;\left(Å\right)=0.88{\left(\frac{p}{p_{0}}\right)}^{2}+6.45\left(\frac{p}{p_{0}}\right)+2.98$$

**Figure 10:**
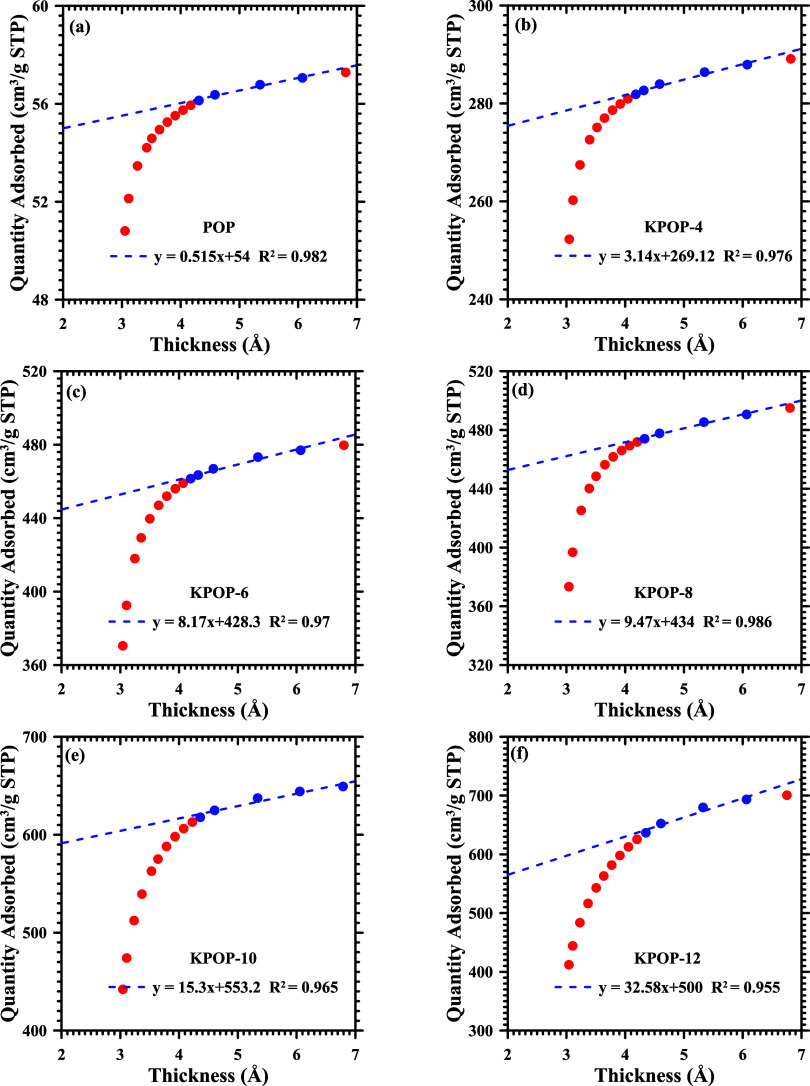
T-plot of the activated carbon: (a) POP, (b) KPOP-4, (c) -6, (d) -8, (e) -10, (f) -10*n*, and (g) -12.

**Figure 11:**
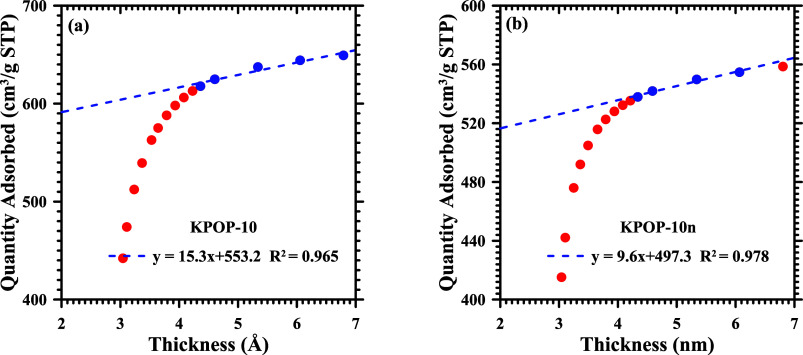
T-plot of the activated carbon: (a) KPOP-10 and (b) KPOP-10*n*.

The micropore surface area is determined by subtracting the external surface area, estimated from the slope of the curve fitting, from the BET surface area. The micropore surface area, micropore to BET surface area ratio, micropore volume, and micropore volume fraction are also listed in Table 3 for comparison. Activated carbon samples exhibit high specific surface areas of micropores (>1700 m^2^/g) and significant micropore volumes (>0.75 cm^3^/g), thereby signifying exceptional microporous characteristics. It is noteworthy that the activation by potassium nitrate significantly enhances both the total pore volume and the micropore surface area while preserving a high proportion of micropores. This observation suggests a well-developed microporous structure that is characterized by partial mesoporosity, which facilitates molecular diffusion and increases functional efficiency.

The pore size distribution graph in Figure 12 illustrates the distribution of pore volume and diameter across all products. It has been observed that the pore volume is positively correlated with the amount of nitrogen adsorption. A higher proportion of potassium nitrate has been found to broaden the range of the pore diameter distribution in the samples, which is consistent with the results observed in the isothermal adsorption–desorption curve. The sample labeled KPOP-10*n*, which did not utilize air assistance, exhibited a significantly smaller pore volume than KPOP-10 and a narrower pore diameter distribution.

**Figure 12:**
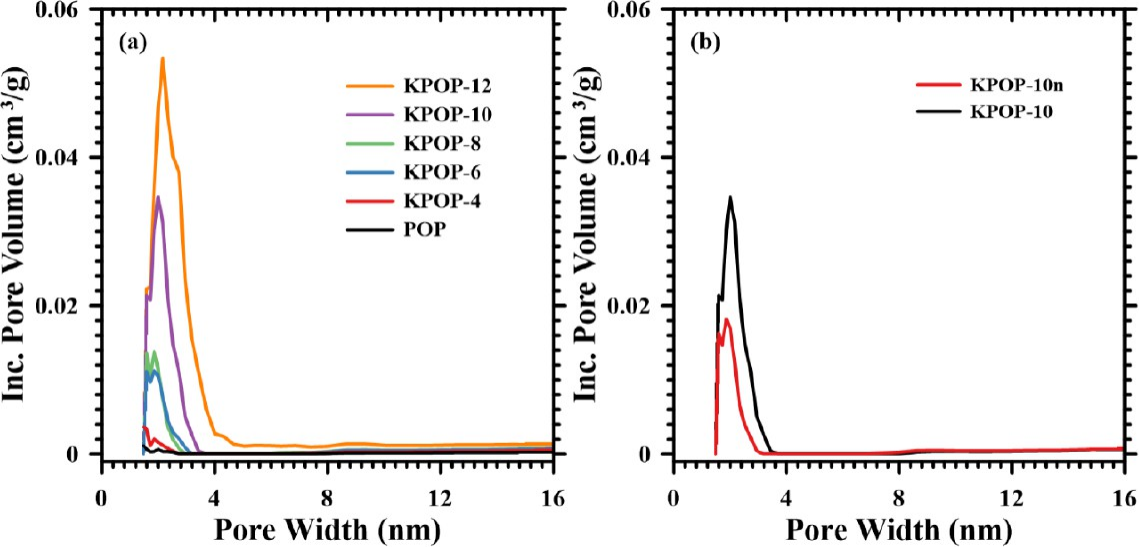
Pore size distribution: (a) POP, KPOP-4, -6, -8, -10 and -12; (b) KPOP-10*n* and -10.

A comparison of the surface area obtained from biomass activated with potassium hydroxide (KOH) is presented in Table 4, providing a benchmark for the current method. The KOH activation technique is well-established for the preparation of activated carbon. According to the results outlined in Table 4, it can be observed that the method employing potassium nitrate demonstrates a comparable performance.

**Table 4:** Comparison of the Surface Area Prepared from Biomass with Activating Agent of KOH

Materials	Activating agent	BET surface area (m^2^/g)	refs
Present study (KPOP-12)	Potassium nitrate	2253	-
Sunflower seed shell	KOH	2509	^53^
Enteromorpha- prolifera	KOH	2283	^54^
Fermented rice	KOH	2106	^55^
Willow catkins	KOH	645	^24^

It is widely recognized that the calcination temperature significantly impacts the carbonization degree of biomasses, the graphitization degree of carbon, and the reaction possibilities and rates for the activating agents and carbon. These factors result in variations in porosity and electrical conductivity. It has been reported that a high calcination temperature induces greater defect formation and enhances the disordered structure of the resultant carbon.^56^ Currently, the calcination temperature for the activation of the activated carbon was set as 800 °C. The methodology employing potassium nitrate, identified as an environmentally friendly agent, is considered novel. Hence, the evaluation of an optimized calcination temperature remains warranted for future studies.

### 3.4 Bonding and Crystallization of Activated Carbon

XRD was employed to detect the structural composition of materials and to assess the presence of impurities based on the positioning of diffraction peaks. The diffraction angles corresponding to graphite crystals are observed at 2θ ≈ 26.5° for the (002) plane and 44.6° for the (101) plane. It is noted that samples subjected to activation, which results in the destruction of their carbon structure, are more likely to exhibit the formation of short-range ordered structures characterized by graphitic microcrystals. This condition leads to the broadening of the diffraction peaks of the samples. As depicted in Figure 13a,b, it is evident that the diffraction peak situated at 2θ between 20° and 30° broadens with the increasing concentration of the potassium nitrate, while some diffraction peaks shift toward more negligible angle scattering, resulting in asymmetrical diffraction peaks. This phenomenon is indicative of a high density of pores within the carbon material.^57^ A thorough examination of the diffraction spectrum reveals that no diffraction peaks attributing to impurities other than carbon materials are detected in any of the samples, thereby providing initial confirmation that significant impurities are absent in the samples.

**Figure 13:**
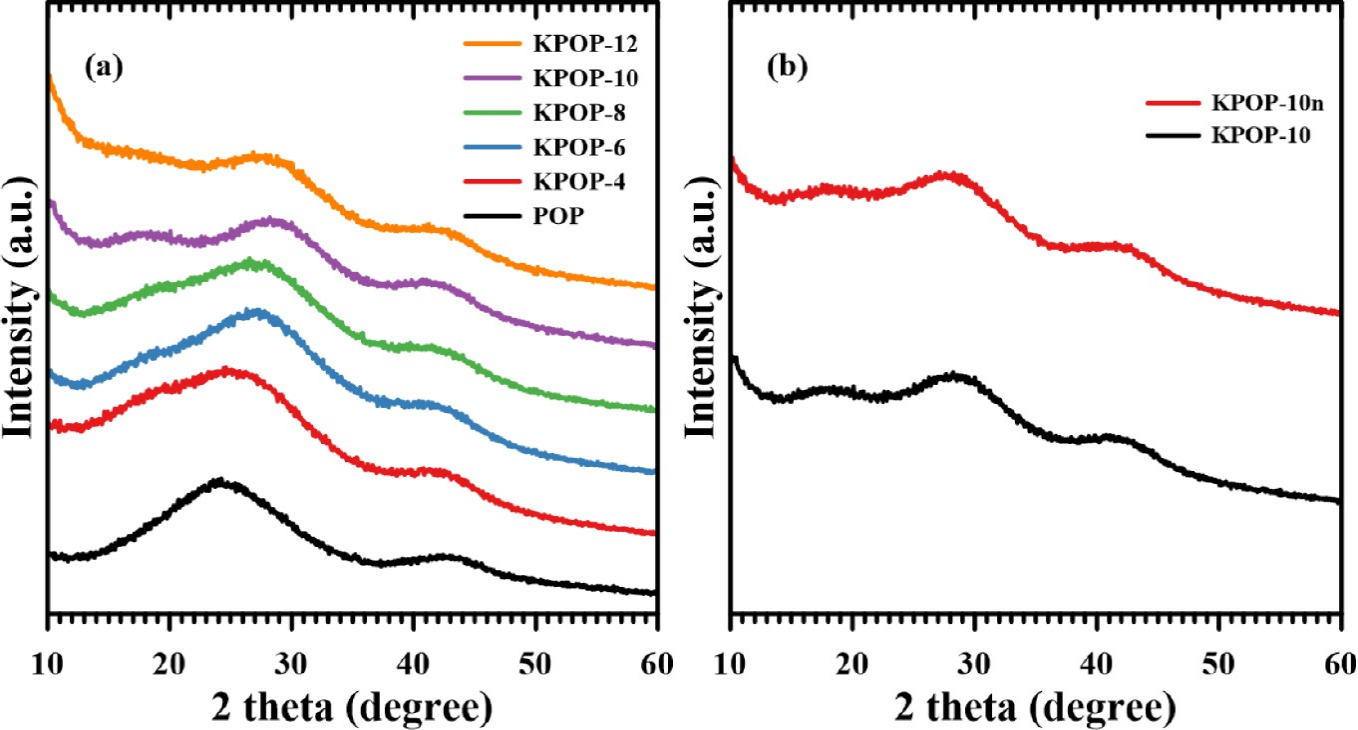
XRD spectrum: (a) POP, KPOP-4, -6, -8, -10 and -12; (b) KPOP-10*n* and -10.

In Figure 14, the Raman spectrum of all samples is presented following peak deconvolution. It should be noted that the D1 band at 1345 cm^–1^ corresponds to the vibrational modes of sp^2^ carbons in the aromatic rings, influenced by in-plane defects and the presence of heteroatoms. Conversely, the G band at 1597 cm^–1^ is linked to the stretching vibration of sp^2^ C–C bonds in a regular polycondensed aromatic structure, indicating the presence of short-range consecutive order of aromatic sheets within the samples. The intensity ratio of the D1 to G band (*I*_D1_/*I*_G_) is observed to correlate positively with the degree of disorder of carbon materials. It has been noted that as the ratio of potassium nitrate increases, the *I*_D1_/*I*_G_ ratio rises from 1.32 in the POP sample to 1.61 in KPOP-12, indicating a positive correlation between the disorder degree of the samples and the quantity of potassium nitrate used. A significant increase in the intensity ratio of the D3 to D4 peaks is observed after the addition of potassium nitrate; these peaks are respectively associated with amorphous carbon and disordered graphite lattices, polyenes, and ionic impurities.^58^

**Figure 14:**
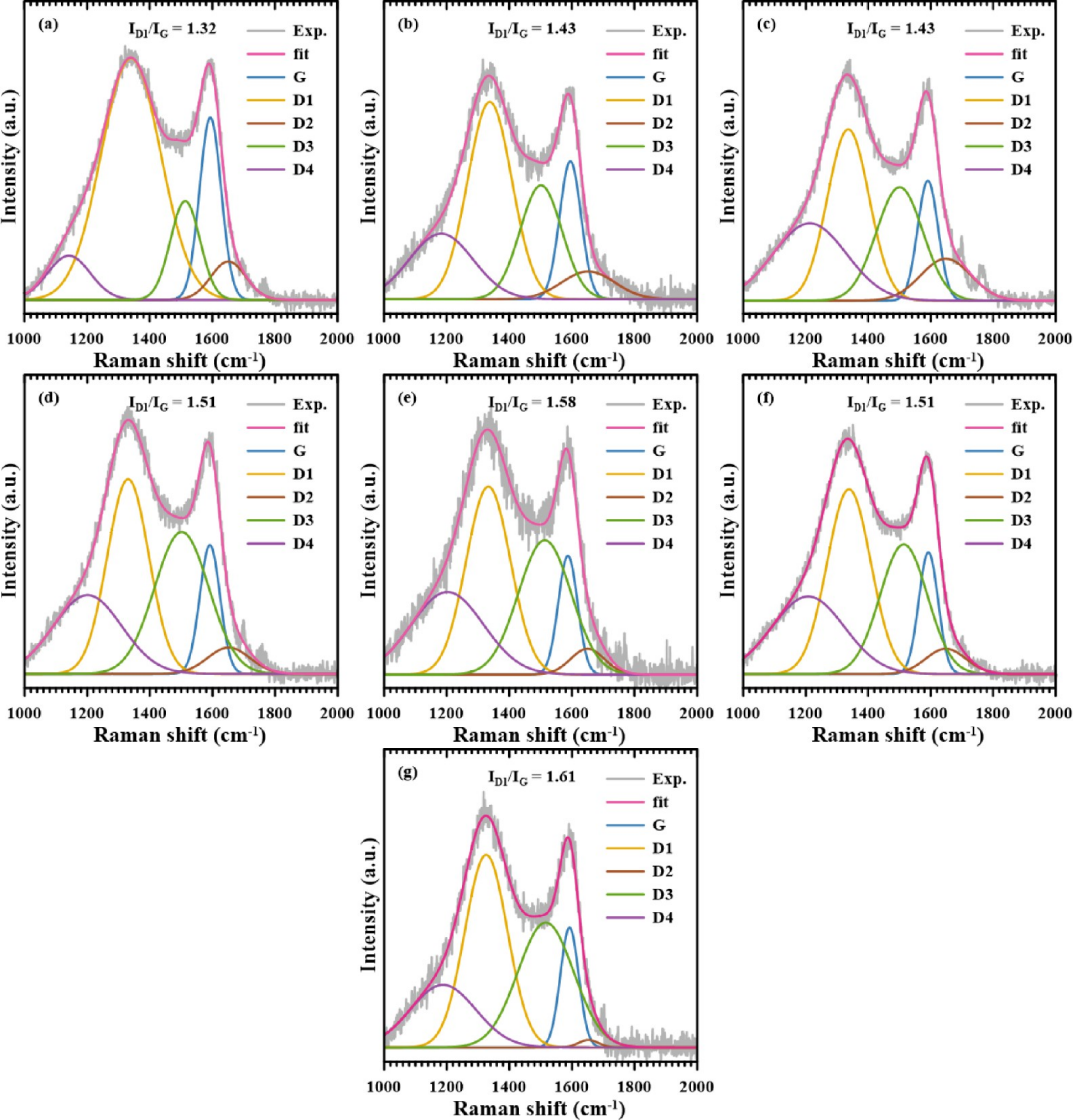
Raman spectrum of the activated carbon: (a) POP, (b) KPOP-4, (c) -6, (d) -8, (e) -10, (f) -10*n*, and (g) -12.

A particularly noteworthy aspect is observed when the current results are compared with findings reported in the literature concerning graphite, graphene oxide, and reduced graphene oxide.^59^ As illustrated in Figure 14, the enhancement in intensity and the shift in the peak position of the D1 band are attributed to the potassium nitrate activation treatment, suggesting a limited fraction of graphene oxide and graphene-type structures within the samples. In the structural analysis of graphene and graphene oxide, the intensity ratio of the D and G bands is used as a criterion to assess the quality of the final product post-treatment.

No diffraction peaks indicative of impurities were found in the previously obtained XRD diffraction patterns, suggesting that the enhancement of the D4 peak is likely attributed to the formation of crystal lattice defects and polyenes during the activation reactions. Based on these observations and the positioning of the G band, it can be concluded that all samples exhibit a graphene structure with nanocrystals. Furthermore, the increased *I*_D1_/*I*_G_ ratio is interpreted as indicative of a higher number of nanocrystals, thereby resulting in an enhanced degree of disorder of the materials.^60,61^

All high-resolution C 1s inverse photoelectron spectra of the samples are presented in Figure 15. It is observed that the carbon atoms within the samples predominantly exhibit bonding configurations of C=C (284.7 eV), C–C (285.3 eV), C–O/C–OH (286 eV), C=O (287 eV), and COOH/COOR (288–290 eV). The diversity in bonding structures of carbon materials is known to influence their physical and chemical properties. A relative ratio table of binding energies and inverse photoelectron peak areas for all samples is provided in Table 5. It can be noted that a slight increase in the total proportion of oxygen-containing functional groups is observed following the introduction of the activation agent into the sample. As the proportion of the activation agent is increased, it is suggested that potassium nitrate initially reacts with disordered carbon and impurity atoms present in the mother phase. Subsequently, a decrease and then an increase in the relative content of C–C bonds within the sample is induced as a consequence of the more extensive destruction of the mother phase structure by the activation agents. Differences in oxygen-containing functional groups, such as C–O/C–OH and COOH/COOR, are noted between KPOP-10 and KPOP-10n. The ratio of carboxylic groups (−COOH) in KPOP-10*n* is observed to be higher than that in KPOP-10, which is believed to adversely affect the application of supercapacitors by hindering the entry of electrolyte ions into the double-layer region, thereby reducing specific capacity and shortening cycle life. Conversely, epoxy groups and hydroxyl groups are recognized for their ability to adsorb solvents and facilitate desolvation, allowing solutes to be closely absorbed onto the electrode surface, which in turn increases charge density per unit area and enhances capacitance. However, it is noted that hydroxyl groups may instigate unstable redox reactions during charging and discharging, ultimately leading to a decrease in the cycling stability of the capacitors.^62^

**Figure 15:**
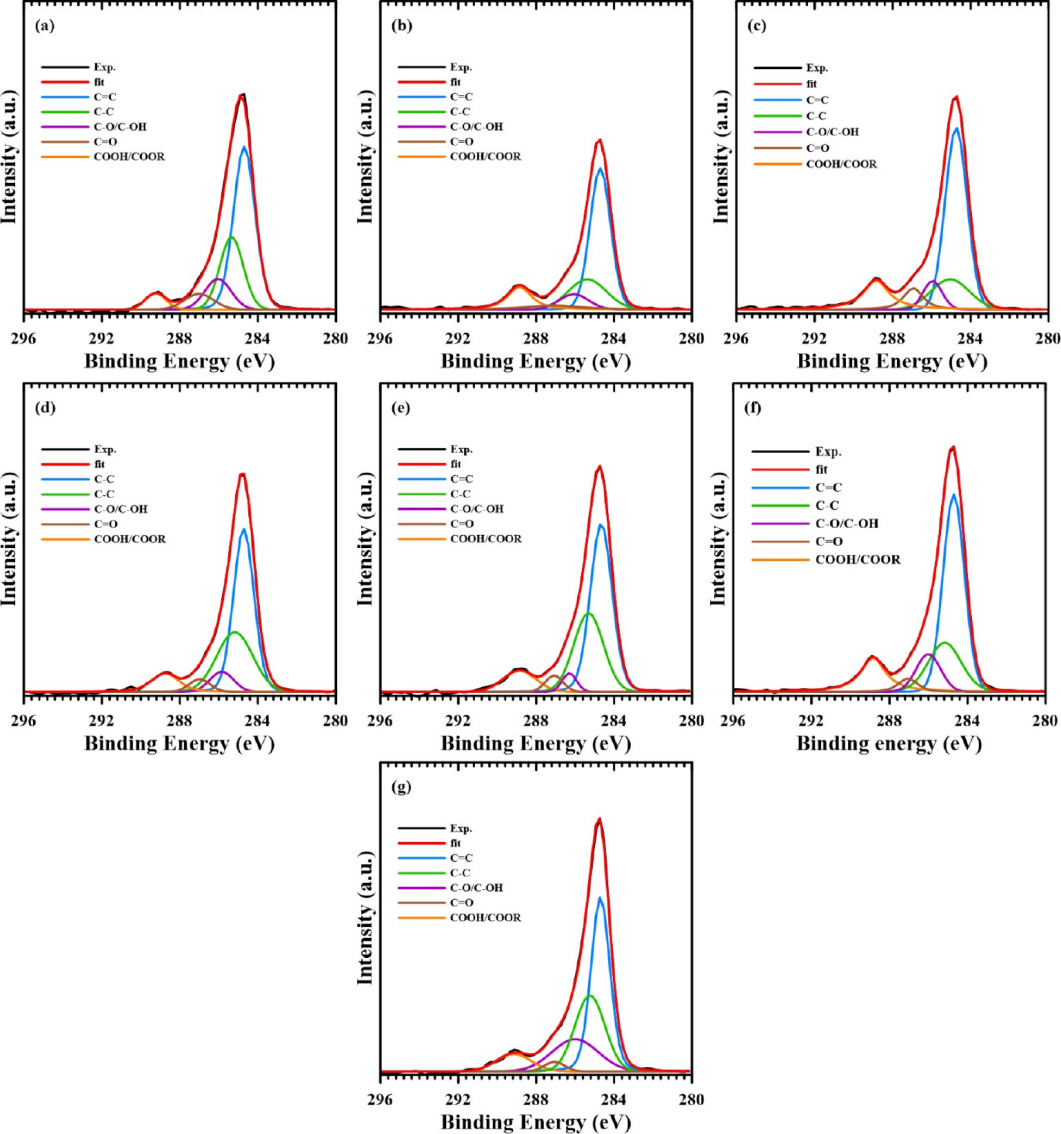
High-resolution C 1s spectra spectrum of XPS analysis: (a) POP, (b) KPOP-4, (c) -6, (d) -8, (e) -10, (f) -10*n*, and (g) -12.

**Table 5:** Relative Proportions of Bonding, Bonding Energies, and Deconvolved Peaks for Activated Carbon

Sample	Bonding species	Binding energy (eV)	Relative area (%)
POP	C=C	284.7	52.91
	C–C	285.4	24.27
	C–O/C–OH	286.1	11.43
	C=O	287	6.57
	COOH/COOR	289.2	4.82
KPOP-4	C=C	284.7	52.88
	C–C	285.4	20.42
	C–O/C–OH	286.1	8.03
	C=O	287.1	5.62
	COOH/COOR	288.9	13.05
KPOP-6	C=C	284.7	53.85
	C–C	285.1	15.76
	C–O/C–OH	285.9	7.49
	C=O	286.9	7.70
	COOH/COOR	288.8	15.20
KPOP-8	C=C	284.7	50.09
	C–C	285.2	30.83
	C–O/C–OH	285.9	6.79
	C=O	287	3.77
	COOH/COOR	288.7	8.52
KPOP-10	C=C	284.7	52.01
	C–C	285.3	31.18
	C–O/C–OH	286.3	3.57
	C=O	287.1	3.87
	COOH/COOR	288.9	9.37
KPOP-10*n*	C=C	284.7	50.40
	C–C	285.2	20.19
	C–O/C–OH	286	10.90
	C=O	287.1	3.80
	COOH/COOR	288.8	14.71
KPOP-12	C=C	284.7	43.86
	C–C	285.2	28.02
	C–O/C–OH	286	18.26
	C=O	287.1	2.38
	COOH/COOR	289.2	7.48

### 3.5 Performance Evaluation of the Fabricated Supercapacitors

In the current study, four samples of activated carbon, designated as KPOP-8, KPOP-10, KPOP-10*n*, and KPOP-12, were selected for the fabrication of supercapacitors due to their superior physical properties. Figure 16a presents the cyclic voltammetry graph of a supercapacitor at a consistent scan rate, indicating that none of the samples exhibited pronounced redox peaks. Notably, KPOP-8 and KPOP-10*n* were observed to display distinct curves when compared to ideal electric double-layer capacitance during the discharging process, with the current increasing gradually as the voltage decreased. This phenomenon may be attributed to high internal resistance and low porosity of the capacitor, which lead to slow diffusion of electrolyte ions. In contrast, the graphs for KPOP-10 and KPOP-12 were more rectangular, suggesting that these capacitors approached electric double-layer behavior during both the charging and discharging processes.

**Figure 16:**
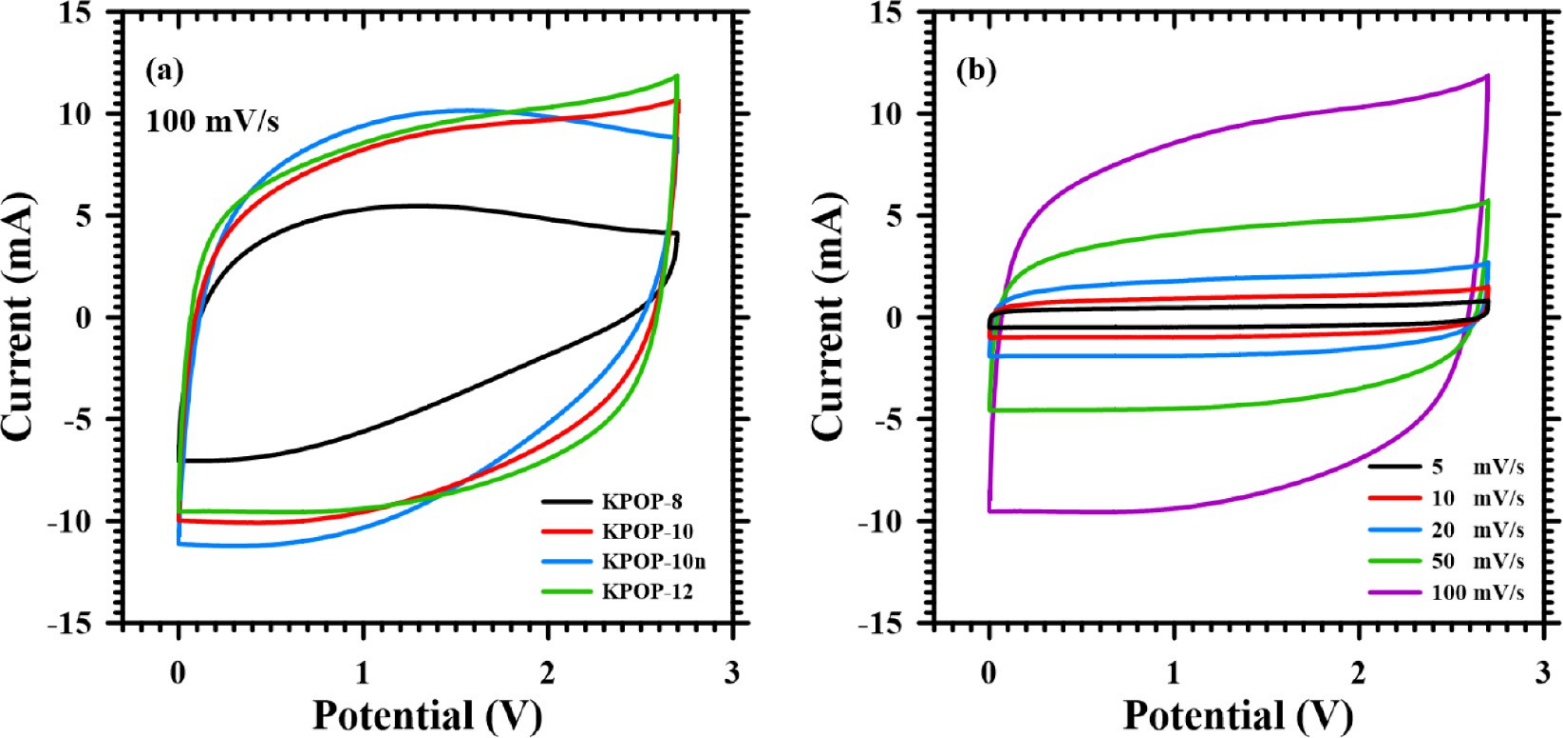
Cyclic voltammetry analysis of the supercapacitors assembled with (a) KPOP-8, -10, -10*n*, and -12 at 100 mV/s and (b) KPOP-12 activated carbon at varied scan rates.

Figure 16b illustrates the cyclic voltammetry graph of the supercapacitor exhibiting the best performance across varying scan rates. It was observed that as the scan rate increased, the diffusion of electrolyte ions was unable to keep pace with the rapid potential conversion, resulting in a delay in the current reaching a constant value upon the reversal of potential scanning. This delay process was found to escalate with increasing scan rate.

The results of cyclic voltammetry analyses of the supercapacitors assembled with the prepared activated carbon are presented in Figure 17. The Nyquist plot for all supercapacitors in this study was scanned from 100 kHz down to 0.1 Hz, and the impedance values obtained across different frequency ranges represent the internal resistance of the system alongside the resistance of the ion diffusion layer. The resistance attributed to the diffusion layer arises from the formation of electric double layers and the limitations imposed by ion diffusion within the electrolyte, which significantly affects the slope of line BC, as illustrated in Figure 17a. When the charging process of a supercapacitor is predominantly governed by the formation of electric double layers, an increase in the slope of line BC is observed; conversely, when ion diffusion plays a more critical role during charging, a decrease in the slope is noted. In Figure 17a, the slopes of line BC for all samples are ranked as KPOP-12 > KPOP-10 > KPOP-10*n* > KPOP-8, indicating that the charging process of sample KPOP-12 is closest to the ideal behavior of an electric double-layer supercapacitor. As the potassium nitrate ratio of the samples decreases, a gradual reduction in the slope is observed, suggesting an increased impact of ion diffusion on the charging process. This phenomenon may be attributed to the influence of pore volume and the range of pore size distribution within the samples. KPOP-8 exhibits the lowest pore volume among the supercapacitors, and its pore size distribution range is relatively narrow, which limits the availability of sufficient mesopores necessary for the effective transmission of electrolyte ions. This limitation results in heightened diffusion resistance of ions, consequently yielding the smallest slope of line BC for KPOP-8 and the highest impedance value. In contrast, the pore volumes and pore size distribution ranges of the remaining three samples increase with the elevation in potassium nitrate ratios, leading to higher slopes of lines BC and diminished charging impedance. An enlarged view of the high-frequency section of the impedance graph from Figure Figure 17a is presented in Figure 17b. Each sample is characterized by a semicircular curve in the high-frequency portion, with points A and B indicating the intersections with the *x*-axis, labeled in Figure 17a. The resistance *R*_A_, corresponding to the *y*-axis at point A, represents the total electrical resistance of both the positive and negative electrodes of the system, while the diameter of the semicircle, defined by the distance between points A and B (*R*_AB_), reflects the electrical resistance of the electrolyte. The internal resistance of the system is derived from the sum of *R*_A_ and *R*_AB_. An analysis of Figure 17b reveals that the internal resistances of KPOP-8 and KPOP-10*n* are closely aligned regarding both electrode and electrolyte resistances. Notably, KPOP-10 demonstrates a lower impedance relative to the electrolyte. XPS results indicate that KPOP-10 possesses a greater number of oxygen-containing functional groups, which facilitate improved wettability and further contribute to the reduction of impedance between the electrodes and the electrolyte.

**Figure 17:**
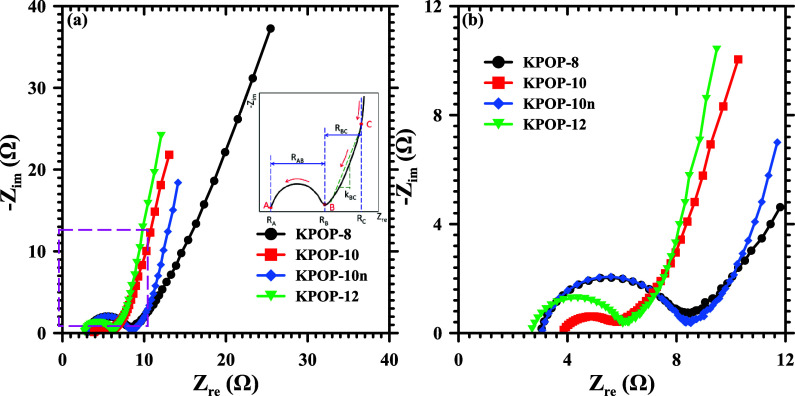
Cyclic voltammetry analysis of the supercapacitors assembled with (a) KPOP-8, -10, -10*n*, and -12 at 100 mV/s and (b) KPOP-12 activated carbon at varied scan rates.

Galvanostatic charge/discharge analyses of the supercapacitors are presented in Figure 18. From Figure 18a, it can be observed that a positive correlation exists between the capacity and the ratio of potassium nitrate addition for different capacitors under the same current density. The voltage–time graphs of four supercapacitors at various current densities (0.5, 1, 2, 5, and 10 A/g) are illustrated in Figure 18b–e. It can be noted from these figures that a shift in the charge and discharge curve of KPOP-8 is evident, which indicates the presence of Faradaic capacitance within the capacitor, alongside minor charging abnormalities occurring at high scanning rates. In the constant current charge and discharge curves of KPOP-8 and KPOP-10n (Figure 18b,d), noticeable voltage drops are observed at low current densities for both electrodes, implying a higher internal resistance of the capacitors. This observation is corroborated by the larger internal resistance indicated in the Nyquist plot of the electrochemical impedance spectroscopy (EIS) for these two samples. The constant current charge and discharge curves of each capacitor are provided in Figure 18. From Figure 18a, a positive correlation between the capacity and the ratio of potassium nitrate addition for different capacitors under the same current density is again evident. The voltage–time graphs illustrating the performance of the supercapacitors at various current densities are shown in Figure 18b–e, reinforce the previous observations regarding the charge and discharge behavior of KPOP-8 and the implications of internal resistance in the analyzed capacitors.

**Figure 18:**
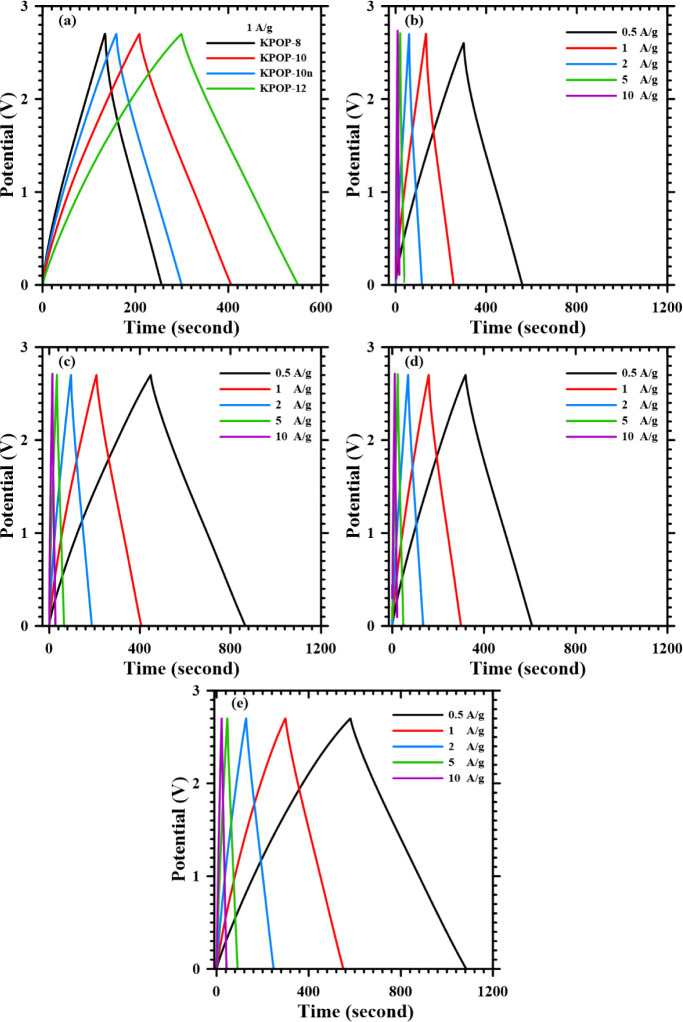
Galvanostatic charge/discharge analysis of the supercapacitors: (a) current density 1 A/g, different electrodes (b) KPOP-8, (c) KPOP-10, (d) KPOP-10*n* and (e) KPOP-12 electrode’s potential-time graph at various current densities.

The specific capacitance of supercapacitors is calculated during the charging and discharging processes. Two primary systems for capacitance measurement are identified, namely the two-electrode and three-electrode systems. The two-electrode system is composed of a working electrode (WE) and a counter/reference electrode (CE/RE), whereas the three-electrode system incorporates a working electrode (WE), a reference electrode (RE), and a counter electrode (CE). The method for calculating the specific capacitance of the two-electrode system is based on the research conducted by Tsay et al.^63^

4 $$\frac{1}{C_{T}}=\frac{1}{C_{p}}+\frac{1}{C_{n}}$$

where *C*_T_ is the total capacitance measured using the two-electrode cell apparatus, *C*_p_ is the capacitance of the positive electrode layer, and *C*_n_ is the capacitance of the negative electrode layer. For asymmetric supercapacitors, it is established that the capacitance values *C*_p_ and *C*_n_ should be equivalent to those of a symmetric supercapacitor. Consequently, the single-electrode capacitance, denoted as *C*, is defined as follows:

5 $$C_{p}=C_{n}=C$$

By substituting eq 3 into eqs 4 and 5 is derived:

6 $$2C_{T}=C$$

The capacitance of a single electrode sheet, *C*_s_, is defined in accordance with eq 6:

7 $$C_{s}=\frac{I\times t}{\frac{\Delta V}{2}}=2C$$

In this equation, *I* represents the current, *t* denotes the charging and discharging time, and Δ*V* signifies the voltage difference. By applying the results of eqs 5 and 6 and subsequently dividing by the weight of the electrode material *m*, the relationship between the specific capacitance of the two-electrode capacitor system (*C*_cp_) and that of a single electrode sheet (*C*_s_) is established, as indicated in eq 7:

8 $$C_{s}=2C=4C_{cp}$$

The energy density and power density of supercapacitors are obtained from the charging and discharging curves, following the formulation presented in eqs 8 and 9. In these equations, *E* represents energy density, *P* denotes power density, *C* signifies specific capacitance, Δ*V* indicates the voltage difference, and Δ*t* refers to the discharge time.^64^

9 $$E=\frac{C\Delta V\times 1000\;g}{2\times 3600\;s}$$

10 $$P=\frac{E\times 3600}{\Delta t}$$

Table 6 presents the specific capacity, energy density, and power density calculated at a current density of 1 A/g for each sample. The best-performing sample, KPOP-12, was found to exhibit a specific capacity of 93.20 F/g and an energy density of 23.59 W h/kg. It was observed that all samples demonstrated a power density of approximately 340 W/kg. Four references were also listed in Table 6 for the performance benchmarking of the supercapacitor assembled in the present study.^65–68^ It is noted that specific capacity, energy density, and power density are all influenced by the assembly process. From the data presented in the table, it can be observed that the power density of the capacitors assembled in this study ranges approximately from 330 to 341 W/kg. Furthermore, the maximum energy density achieved is 23.59 W h/kg, which is considered reasonable and comparable to the findings of other studies.

**Table 6:** The Specific Capacity, Energy Density, and Power Density Calculated at a Current Density of 1 A/g

Sample	Specific capacitance (F/g)	Single electrode specific capacitance (F/g)	Energy density (W h/kg)	Power density (W/kg)	Remark
KPOP-8	11.29	45.16	11.43	340.07	This work
KPOP-10	18.38	73.53	18.61	336.66	This work
KPOP-10*n*	13.11	52.45	13.27	341.23	This work
KPOP-12	23.30	93.20	23.59	338.34	This work
Material/activation process					
Saw dust/KOH-hydrothermal process	75.6	303	17.75	436	^65^
Sugar cane bagasse/KOH-hydrothermal	70	280	7	571	^66^
Rubber wood/CO_2_	34.5	138	2.63	291	^67^
Garlic peels/thermal	43.5	174	32.6	108	^68^

Recent studies have indicated that utilizing activated carbon alone in the fabrication of supercapacitors has gradually encountered a bottleneck.^69–71^ Most research efforts regarding the preparation of activated carbon have focused on environmentally friendly synthesis methods. To address the demands of modern energy storage, investigations into alternative electrode materials, such as perovskite,^69^ molybdenum boride,^70^ and iron–molybdenum carbide bimetallics,^71^ have been proposed. The energy density and power density of these supercapacitors have been reported to reach values of 54.6 W h/kg and 850 W/kg, respectively.^71^ Within the packaging of supercapacitors, carbon black or activated carbon continues to hold significant importance; thus, developing superior methods for preparing carbon black and activated carbon remains a topic of considerable research significance.

## 4 Conclusions

The preparation of high porosity activated carbon from porous biomass using potassium nitrate and its application in electric double-layer supercapacitors was experimentally studied. Key findings include:

1. SEM and TEM analyses show that potassium nitrate-activated samples develop porosity and flaky structures due to self-structuring and vigorous thermal reactions during activation.
2. XRD and Raman spectroscopy reveal that the activated carbon consists of graphite crystals interspersed with disordered carbon. A notable (002) diffraction peak indicates that potassium nitrate promotes high-density pore formation.
3. Nitrogen adsorption–desorption isotherm results demonstrate that the majority of the activated carbon contains micropores and narrow mesopores. An increase in activator ratio correlates positively with specific surface area and pore volume.
4. Elemental analysis shows that higher activator ratios lead to increased carbon content in the activated carbon. The KPOP-10n sample, activated in a nitrogen atmosphere, exhibits the highest carbon content, highlighting the influence of activation conditions.
5. XPS analysis demonstrates that samples contain C, O, and N, with C–C bond content fluctuating based on activator levels. An optimal amount of potassium nitrate clears blocked pores, while excess disrupts the structure.
6. The supercapacitor constructed with KPOP-12 displays the highest specific capacitance and energy density, with EIS analysis confirming its close alignment with ideal supercapacitor charging behavior.

## Notes

Declaration of Generative AI and AI-Assisted Technologies in the Writing Process: During the preparation of this work, the authors wrote all the draft documents. The authors used Grammarly in order to fix all grammatical and spelling errors and improve readability ONLY. After using the tool, the authors reviewed and edited the content as needed and will take full responsibility for the content of the publication.

## Nomenclature

Symbols: Description unit
C: capacitance, F
E: Energy density, W h/kg
I: Current, A
M: Weight; mass, g
P: Power density, W/kg
p: pressure, bar
R: Resistance, ohm
t: time, s
V: voltage, volt
Y: Mass fraction
**Greek**
Δ: difference
ξ: Statistical thickness
χ: rate
**Subscripts**
ac: Activated carbon
b: Biomass
c: Carbon
cc: Carbon conversion
cp: Capacitor
n: Negative electrode
o: saturation pressure of the adsorbate gas
p: Positive electrode
s: Single electrode
T: Total
y: Yield
